# Insulin-like peptide 5 is a microbially regulated peptide that promotes hepatic glucose production

**DOI:** 10.1016/j.molmet.2016.01.007

**Published:** 2016-01-25

**Authors:** Ying Shiuan Lee, Filipe De Vadder, Valentina Tremaroli, Anita Wichmann, Gilles Mithieux, Fredrik Bäckhed

**Affiliations:** 1 The Wallenberg Laboratory, Department of Molecular and Clinical Medicine, University of Gothenburg, 41345 Gothenburg, Sweden; 2 Institut National de la Santé et de la Recherche Médicale, U855, Lyon 69372, France; 3 Université de Lyon, Lyon 69008, France; 4 Université Lyon 1, Villeurbanne 69622, France; 5 Novo Nordisk Foundation Center for Basic Metabolic Research, Section for Metabolic Receptology and Enteroendocrinology, Faculty of Health Sciences, University of Copenhagen, 2200 Copenhagen, Denmark

**Keywords:** Insulin-like peptide 5 (INSL5), Gut microbiota, Liver, Colon

## Abstract

**Objective:**

Insulin-like peptide 5 (INSL5) is a recently identified gut hormone that is produced predominantly by L-cells in the colon, but its function is unclear. We have previously shown that colonic expression of the gene for the L-cell hormone GLP-1 is high in mice that lack a microbiota and thus have energy-deprived colonocytes. Our aim was to investigate if energy deficiency also affected colonic *Insl5* expression and to identify a potential role of INSL5.

**Methods:**

We analyzed colonic *Insl5* expression in germ-free (GF), conventionally raised (CONV-R), conventionalized (CONV-D) and antibiotic-treated mice, and also assessed the effect of dietary changes on colonic *Insl5* expression. In addition, we characterized the metabolic phenotype of *Insl5*−/− mice.

**Results:**

We showed that colonic *Insl5* expression was higher in GF and antibiotic-treated mice than in CONV-R mice, whereas *Insl5* expression in the brain was higher in CONV-R versus GF mice. We also observed that colonic *Insl5* expression was suppressed by increasing the energy supply in GF mice by colonization or high-fat feeding. We did not observe any differences in food intake, gut transit or oral glucose tolerance between *Insl5*−/− and wild-type mice. However, we showed impaired intraperitoneal glucose tolerance in *Insl5*−/− mice. We also observed improved insulin tolerance and reduced hepatic glucose production in *Insl5*−/− mice.

**Conclusions:**

We have shown that colonic *Insl5* expression is regulated by the gut microbiota and energy availability. We propose that INSL5 is a hormone that could play a role in promoting hepatic glucose production during periods of energy deprivation.

## Highlights

• INSL5 is a microbially regulated peptide hormone.
• Colonic INSL5 expression is suppressed by increased energy availability.
• INSL5 promotes hepatic glucose production.

## 1 Introduction

Insulin-like peptide (INSL) 5 is a member of the relaxin/insulin family [1], which comprises insulin, insulin-like growth factor (IGF) 1 and 2 [2], [3], [4], relaxin 1 and 2, and INSL3-7 [5], and has been recently identified in colonic and brain tissue [1], [6], [7], [8]. Although other members of the relaxin/insulin family are known to have roles in glucose metabolism, reproductive physiology and remodeling of connective tissue [5], [9], [10], [11], [12], the function of INSL5 is not clear. One study based on observations in *Insl5*−/− mice indicated that INSL5 may regulate glucose metabolism by affecting pancreatic beta cell number, but the *Insl5*−/− phenotype was mild and dependent on the genetic background of the mice [13]. Another study reported that INSL5 enhances glucose-stimulated insulin secretion, both *in vivo* and *in vitro* [14]. A more recent study suggested that INSL5 is an orexigenic gut hormone that is upregulated after fasting and calorie restriction [15]. In summary, these studies suggest that INSL5 may have a role in regulation of host energy metabolism.

The gut microbiota is known to contribute to efficient energy harvest from the diet by degrading plant polysaccharides, such as cellulose, xylan, pectin and resistant starch [16], [17], [18], and to promote energy storage by modulating the expression of host genes [19]. The microbially produced short-chain fatty acid (SCFA) butyrate is the primary energy source for colonocytes, and thus germ-free (GF) mice (i.e. mice that lack a microbiota) have energy-deprived colonocytes [20]. We recently reported that a lack of microbiota reduced the energy availability in the colon which increased the expression of *Gcg* [the gene for proglucagon, the precursor of glucagon-like peptide-1 (GLP-1)], and proposed that colonic GLP-1 plays an important role in slowing intestinal transit under conditions of calorie restriction [21]. A previous microarray screen of tissues from GF and conventionally raised (CONV-R) mice revealed that colonic *Insl5* expression is significantly regulated by the microbiota [22]. Because both GLP-1 and INSL5 are secreted from colonic enteroendocrine L-cells, we hypothesized that colonic *Insl5* expression is similarly modulated by energy availability.

Here we investigated how the gut microbiota and energy deficiency affect the colonic expression of *Insl5* and used *Insl5*−/− mice to explore the role of INSL5. We provide evidence that INSL5 plays a role in promoting hepatic glucose production during periods of fasting.

## 2 Material and methods

### 2.1 Mice and diets

GF Swiss Webster and C57Bl/6J mice were maintained in flexible film isolators under a strict 12-hour light cycle. GF status was monitored regularly by anaerobic culturing and PCR for bacterial 16S rRNA. *Insl5*−/− mice on a C57Bl/6J background (Deltagen Target ID#65) were purchased from Jackson Deltagen (San Mateo, CA, USA), backcrossed with our C57Bl/6J mice for >10 generations, and bred in our facility to harmonize the gut microbiota. Thereafter, C57Bl/6 WT and *Insl5*−/− mice were maintained as separate colonies.

Unless otherwise indicated, experiments were performed with male mice aged 12–14 weeks that were fed an autoclaved low-fat polysaccharide-rich chow diet (LabDiet, St Louis, MO, USA) *ad libitum*. For high-fat diet experiments, mice were weaned onto a high-fat, high-sugar western diet with 40% of calories from fat (Adjusted Fat Diet TD.96132, Harlan Teklad, Indianapolis, IN, USA). At the end of the experiments and unless otherwise indicated, mice were fasted for 4 h before being killed, and organs were harvested and flash-frozen in liquid nitrogen. All mouse experiments were performed using protocols approved by the Research Animal Ethics Committee in Gothenburg, Sweden.

### 2.2 Colonization of GF mice

For colonization with an unfractionated microbiota, GF mice were colonized with total cecal content from a CONV-R donor. The cecal content was resuspended in 5 ml sterile PBS and 200 μl of cecal slurry was given by oral gavage to each GF mouse. The resulting conventionalized (CONV-D) mice were kept in standard cages for 1, 3 or 7 days. For monocolonization experiments, *Bacteroides thetaiotaomicron* VPI-5482 (ATCC 29148) in liquid culture was fed to GF mice. Monocolonized mice were housed in separate sterile isolators for 4 weeks. At the end of the colonization period, mice were fasted for 4 h before killing and tissue harvest. Colonization density by *B. thetaiotaomicron* was verified by culture enumeration.

### 2.3 Antibiotic treatment

A cocktail of bacitracin, neomycin, and streptomycin (200 mg/kg bodyweight of each antibiotic) (Sigma Aldrich, St Louis, MO, USA) or water (vehicle control) was given by oral gavage to mice daily for 3 days. Colonic tissue from the mice was analyzed on day 4.

### 2.4 Quantitative RT-PCR (qRT-PCR)

Mouse tissues were homogenized in RLT buffer supplemented with 2-mercaptoethanol using 5 mm steel beads and TissueLyser (Qiagen, Hilden, Germany). RNA was isolated using the RNeasy Kit with on-column DNase I treatment (Qiagen). cDNA was synthesized from the total RNAs using the High Capacity cDNA Reverse Transcription Kit (Applied Biosystems, Foster City, CA, USA) according to the manufacturer's manual. qRT-PCR reactions were prepared in a 25 μl volume containing 1× SYBR Green Master Mix buffer (Thermo Scientific, Waltham, MA, USA) and 900 nM specific primers targeting gene of interest (or 300 nM directed against the L32 gene expression). Reactions were run on a CFX96 Real-Time System (Bio-Rad, Hercules, CA, USA). Gene expression data were normalized to the expression level of the ribosomal protein L32 using the ΔΔC_T_ method and analyzed by calculating relative gene expression. Primer sequences are listed in Supplementary Table 1.

### 2.5 Immunohistochemistry

Colon tissues were fixed in 4% paraformaldehyde in PBS for 24 h and washed and dehydrated with ethanol. Paraffin-embedded sections (8 μm) were prepared. For staining, sections were deparaffinized and exposed to antigen unmasking in antigen retrieval 2100 using 10 mM sodium citrate buffer pH 6.0. After rinsing, sections were incubated in blocking buffer (10% goat serum, 1% bovine serum albumin and 0.1% Triton X-100 in PBS) for 1 h at room temperature. Sections were stained with anti-GLP-1 mouse monoclonal subtype IgG1 antibody (ab26278, Abcam, Cambridge, UK) diluted 1:400 or anti-peptide YY (PYY) chicken polyclonal antibody (ab15879, Abcam) diluted 1:800, and anti-INSL5 rabbit polyclonal antibody (G-035-40, Phoenix Pharmaceuticals, Burlingame, CA, USA) diluted 1:200 in blocking buffer overnight at 4 °C. Primary antibodies were targeted with immunofluorescent dye labeled secondary antibodies Alexa Fluor 568 anti-mouse IgG1 (γ1) (A21124) or Alexa Fluor 594 Goat anti-chicken (A11042) and Alexa Fluor 488 Goat anti-rabbit IgG (A11008), all diluted 1:1000 (Life Technologies, Carlsbad, CA, USA). Cell nuclei were counterstained with Hoechst 33342 nucleic acid stain (H1399, Life Technologies).

### 2.6 Measurements of body weight, total body fat content and food intake

WT and *Insl5*−/− mice were weaned on to standard chow diet at 3 weeks of age and were weighed once a week. For total body fat measurements, mice were anesthetized with isoflurane gas and dual-energy X-ray absorptiometry (DEXA) was performed by using the small animal densitometer (Lunar PIXImus Mouse, GE Medical Systems, Waukesha, WI, USA). Food intake was measured over 1 h in mice subjected to a previous 12 h fast.

### 2.7 Upper GI transit

WT and *Insl5*−/− mice were fasted overnight with *ad libitum* access to water. In the morning, mice were gavaged with 200 μl of 1.5% methylcellulose containing 5% Evans blue dye (Sigma–Aldrich). After 15 min, mice were killed, and the intestine from the region of the pyloric sphincter to the ileo-caecal junction was removed. The gut transit is presented as the distance the Evans blue dye traveled as a percentage of the whole length of the small intestine.

### 2.8 Oral and intraperitoneal glucose tolerance tests

Mice were fasted for 6 h and given either an oral gavage of glucose (2 g/kg body weight) or an intraperitoneal injection of glucose (1 g/kg body weight). Tail blood was collected and blood glucose measured with HemoCue 201+ analyzer (HemoCue, Ängelholm, Sweden) before (30 and 0 min) and after (15, 30, 60, 90 and 120 min) gavage or injection. Tail blood was also collected with Microvette CB 300 Z (Sarstedt, Nümbrecht-Rommelsdorf, Germany) for serum insulin analysis using the Ultra-Sensitive Mouse Insulin ELISA kit (Crystal Chem, Downers Grove, IL, USA) according to the manufacturer's protocol.

### 2.9 Insulin tolerance test and pyruvate tolerance test

Tolerance tests were performed in WT and *Insl5*−/− mice given an intraperitoneal injection of insulin (0.75 U/kg bodyweight after a 6 h fast) or pyruvate (2 g/kg bodyweight after a 12 h fast). Glucose was measured in tail blood samples as described above.

### 2.10 Immunoblot analysis

Frozen liver tissues were homogenized and immunoblotting was performed using rabbit anti-G6PC (custom made) dilution 1:5000 and rabbit anti-PEPCK (H-300) dilution 1:7000 (sc-32879) (Santa Cruz Biotechnology, Dallas, TX, USA) and normalized to the housekeeping gene tubulin (9F3) dilution 1:1000 (2128) (Cell Signaling, Danvers, MA, USA). The primary antibodies were detected with corresponding secondary HRP-conjugated goat anti-rabbit IgG (170-5046) (Biorad, Hercules, CA, USA) antibodies (dilution 1:10,000). The signal was quantified using the software ImageJ.

### 2.11 G6Pase activity assay

Frozen liver tissues were ground to fine powder with a stainless steel mortar at liquid nitrogen temperature. The powder was homogenized in 10 mM HEPES and 0.25 M sucrose, pH 7.4 (9 vol/g tissue) by ultrasonication. G6Pase activity was directly assayed in homogenates for 10 min at 30 °C at pH 7.3 under maximal velocity conditions under the presence of saturated glucose-6-phosphate concentration (20 mM). Non-specific phosphatase activity was also determined by preparation of additional samples containing β-glycerophosphate. Specific activity of G6Pase was obtained by subtraction of non-specific phosphatase activity (after hydrolysis of β-glycerophosphate) from specific activity obtained by glucose-6-phosphate hydrolysis [23].

### 2.12 Glycogen measurements

Frozen liver tissues were ground to fine powder at liquid nitrogen temperature. Liver tissues were extracted with 6% perchloric acid and adjusted to pH 6.5–8.5 with 3.2 M K_2_CO_3_. Liver glycogen levels were measured with the α-amyloglucosidase method [24]. Tissues were harvested at 1PM (note that lights are turned on at 7AM).

### 2.13 Statistical analysis

Data are presented as mean ± SEM. Statistical differences between two groups were analyzed with a Student's t test. Comparisons of three or more groups with one independent variable were analyzed by one-way ANOVA with *post hoc* Bonferroni test.

## 3 Results

### 3.1 Colonic *Insl5* expression is downregulated by the microbiota and by energy availability

**Figure 1:**
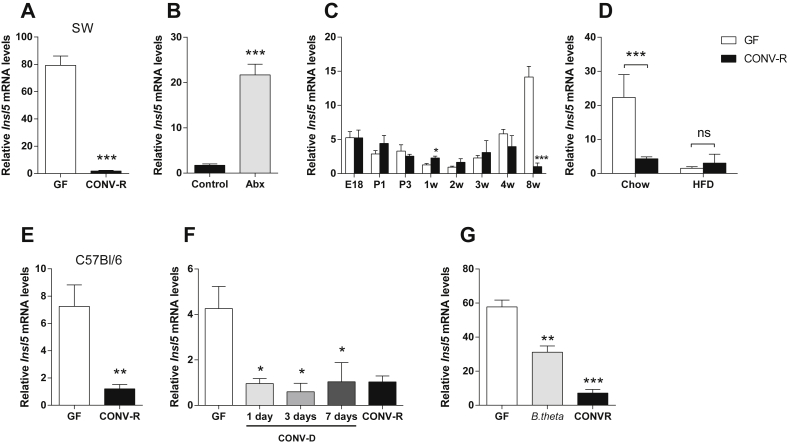
**Colonic *Insl5* expression is reduced by the gut microbiota and energy availability**. *Insl5* expression in colon from **(A)** Swiss Webster GF and CONV-R mice (*n* = 4–5), **(B)** Swiss Webster mice after 3 days of antibiotic treatment (Abx) or control (*n* = 4–6), **(C)** Swiss Webster GF and CONV-R mice on embryonal day 18 (E18), postnatal days 1 (P1) and 3 (P3) and during weeks 1–8 of their life (1 w–8 w) (*n* = 5), **(D)** Swiss Webster GF and CONV-R mice on a standard chow diet or a high-fat diet (HFD) (*n* = 4–6), **(E)***Insl5* expression in colon from C57Bl/6 GF and CONV-R mice (*n* = 4–5), **(F)** C57Bl/6 GF, CONV-D (GF mice that were conventionalized with a normal gut microbiota for 1, 3 and 7 days) and CONV-R mice (*n* = 3–4), and **(G)** C57Bl/6 GF, *B. thetaiotaomicron*-colonized and CONV-R mice, (*n* = 3–4). Data are mean ± SEM. *p < 0.05, **p < 0.01, ***p < 0.001. In F, samples were analyzed by one-way ANOVA with *post hoc* Bonferroni test, where the mean of each test group was compared to the mean of the (GF) control group.

A recent microarray-based screen to identify microbially regulated genes in different tissues showed that *Insl5* is one of the most significantly regulated genes in the colon [22]. We confirmed this microbial regulation by qRT-PCR and showed that expression of *Insl5* mRNA was 80-fold higher in the colon of GF compared with CONV-R Swiss Webster mice (Figure 1A). To investigate whether colonic *Insl5* expression could also be induced by reducing the bacterial load in CONV-R mice, we treated Swiss Webster mice with antibiotics for three days and found that the treatment cocktail resulted in a 20-fold increase in *Insl5* expression (Figure 1B).

In the absence of a microbiota, colonocytes are energy deficient because of a lack of their main energy source, namely microbially produced SCFAs [20]. Because we previously observed that colonic expression of *Gcg* (the gene from which GLP-1 is derived) is negatively regulated by the microbiota and by energy availability [21], we hypothesized that colonic *Insl5* expression is upregulated in GF mice because of reduced energy levels in the GF colon. First, we tested this hypothesis by investigating differences in colonic *Insl5* expression in GF and CONV-R mice before and after weaning, a biologically important energy transition when the pups' diet is changed from energy-dense milk (which provides energy to both CONV-R and GF colonocytes in newborn mice) to standard chow (which is rich in plant polysaccharides and only metabolized to SCFAs in the presence of a microbiota). In agreement with our hypothesis, we observed little or no difference in colonic *Insl5* expression between GF and CONV-R Swiss Webster mice before or shortly after weaning (at 3 weeks), but we observed a substantial (15-fold) increase in colonic *Insl5* expression in GF mice at 8 weeks of age (Figure 1C). To further investigate whether increased energy supply from the diet could suppress the elevated colonic *Insl5* expression in GF mice, we analyzed colonic *Insl5* expression in GF and CONV-R Swiss Webster mice 1 week after they were weaned onto a standard chow diet (which is low in fat) or an energy-rich high-fat diet (40% of calories from fat). Although we observed significantly higher colonic *Insl5* expression in GF compared with CONV-R mice on a chow diet, this difference was abolished in mice fed a high-fat diet, and colonic *Insl5* expression was low in high-fat-fed mice regardless of bacterial status (Figure 1D).

We also confirmed that colonic *Insl5* expression was significantly higher in GF compared with CONV-R C57Bl/6 mice (Figure 1E). To explore the kinetics of microbial suppression of *Insl5* expression, we colonized GF C57Bl/6 mice with microbiota obtained from a CONV-R mouse cecum (conventionalized; CONV-D) and found that the *Insl5* expression was suppressed to levels similar to those observed in CONV-R mice within 1 day of colonization (Figure 1F). We also colonized GF C57Bl/6 mice with *B. thetaiotaomicron*, a Gram-negative bacterium that ferments a wide range of plant polysaccharides [25] and increases the levels of acetate and propionate upon colonization [21], and showed that *B. thetaiotaomicron* reduced the colonic *Insl5* expression by 50% (Figure 1G).

Together, these results show that colonic *Insl5* expression is suppressed by the presence of a gut microbiota and increased energy availability in both Swiss Webster and C57Bl/6 mice. We observed that microbial regulation of the *Insl5* expression is similar to *Gcg*, and immunohistochemical analysis confirmed that INSL5 is produced by colonic L-cells, which also produce GLP-1 and PYY (Supplementary Figure 1A and B).

### 3.2 *Insl5* expression in the brain is upregulated by the microbiota

**Figure 2:**
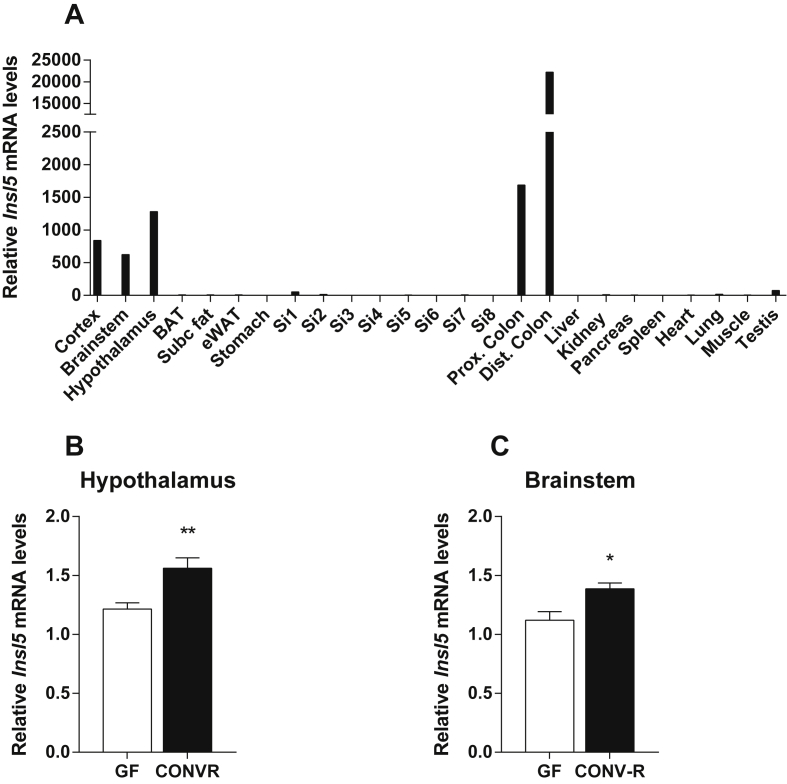
***Insl5* expression is detected in the colon and the brain**. **(A)***Insl5* expression in different C57Bl/6 mouse tissues (samples are pooled from *n* = 3). Si = small intestine; the numbers indicate that the small intestine was divided in eight equal sized pieces labeled 1 (duodenum) to 8 (ileum). *Insl5* expression in **(B)** hypothalamus (*n* = 13) and **(C)** brainstem from C57Bl/6 GF and CONV-R mice (*n* = 5–7). Data are mean ± SEM. *p < 0.05, **p < 0.01.

In agreement with previous studies [1], [6], [7], [8], [15], we showed that *Insl5* exhibited highest expression in the distal colon, but was also expressed in the proximal colon and the brain (Figure 2A). To investigate whether the gut microbiota also decreased expression of *Insl5* in the brain, we analyzed *Insl5* expression in the hypothalamus and brainstem of GF and CONV-R C57Bl/6 mice. However, in contrast to the colon, we observed lower expression of *Insl5* in these brain regions in GF compared with CONV-R mice (Figure 2B,C).

### 3.3 *Insl5*−/− mice have impaired hepatic glucose production

To investigate the role of INSL5 *in vivo*, we obtained *Insl5*−/− mice on a C57Bl/6 background. *Insl5*−/− mice were born at the expected Mendelian ratio, exhibited normal gross appearance, growth and fat composition (Supplementary Figure 2A–C), and were fertile and produced normal offspring. We did not observe any differences in food intake or gut transit, physiological functions modulated by gut hormones [26], [27], between *Insl5*−/− and WT mice (Supplementary Figure 2D,E).

**Figure 3:**
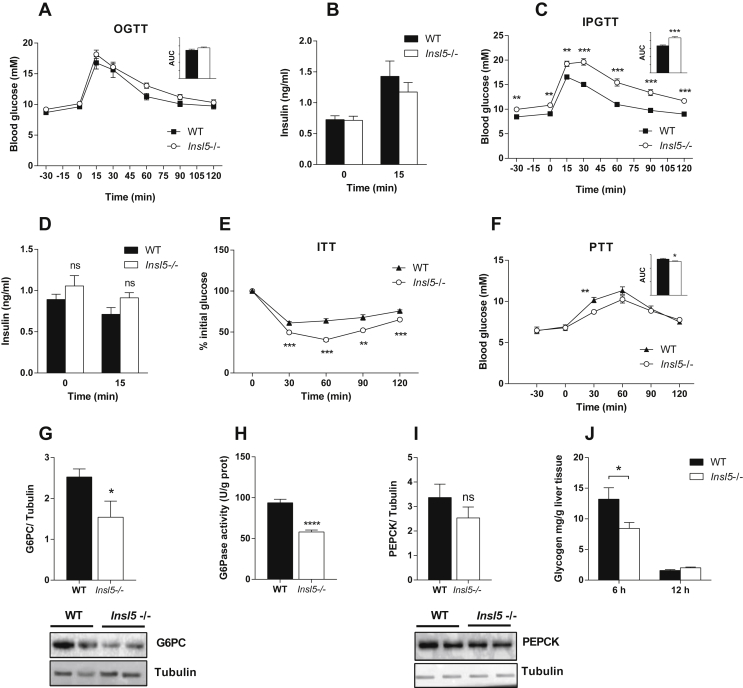
**C57Bl/6 *Insl5*−/− mice have impaired** hepatic glucose production. **(A)** Oral glucose tolerance test (OGTT) and **(B)** serum insulin levels after glucose gavage in 12 to 14-week-old C57Bl/6 WT and *Insl5*−/− mice (*n* = 7–24). **(C)** Intraperitoneal glucose tolerance test (IPGTT) and **(D)** serum insulin levels after glucose injection in *Insl5*−/− and C57Bl/6 WT mice (*n* = 10). **(E)** Insulin tolerance test (ITT) in *Insl5*−/− and C57Bl/6 WT mice (*n* = 7). **(F)** Pyruvate tolerance test (PTT) in *Insl5*−/− and C57Bl/6 WT mice (*n* = 9–10). **(G)** Immunoblot analysis of G6PC in liver tissue from *Insl5*−/−mice and C57Bl/6 WT mice after a 12 h fast (*n* = 8–9). **(H)** G6Pase activity in liver tissue from C57Bl/6 *Insl5*−/−mice and WT mice after a 12 h fast (*n* = 6–9). **(I)** Immunoblot analysis of PEPCK in liver tissue from C57Bl/6 *Insl5*−/−mice and WT mice after a 12 h fast (*n* = 8–9). **(J)** Glycogen levels in liver tissue from *Insl5*−/−mice and C57Bl/6 WT mice after a 6 and 12 h fast (*n* = 6–9). Data are mean ± SEM. *p < 0.05, **p < 0.01, ***p < 0.001.

To assess the potential effects of INSL5 on glucose homeostasis, we performed glucose tolerance tests in *Insl5*−/− and WT mice after a 6 h fast. Orally administered glucose increased blood glucose and insulin levels to a similar extent in *Insl5*−/− and WT mice (Figure 3A,B). In contrast, glucose tolerance after an intraperitoneal glucose injection was impaired in *Insl5*−/− compared with WT mice (Figure 3C), in agreement with a previous finding [13], although no differences in blood insulin were observed between the two groups of mice (Figure 3D). We noted that fasting glucose levels in *Insl5*−/− mice were higher than in WT mice before the intraperitoneal glucose tolerance test (Figure 3C) but not before the oral glucose tolerance test (Figure 3A). However, we showed that differences in glucose tolerance after an intraperitoneal glucose injection between *Insl5*−/− and WT mice remained significant when the data were normalized to baseline levels of glucose (Supplementary Figure 2F).

Surprisingly, insulin tolerance was improved in *Insl5*−/− compared with WT mice (Figure 3E). We therefore speculated that *Insl5*−/− mice have a delayed ability to recover from low blood glucose levels 30 min after an insulin injection by failing to promote a counter-regulatory induction of glucose production. To test this hypothesis, we performed a pyruvate tolerance test to investigate whether glucose production from pyruvate, a major substrate of hepatic gluconeogenesis [28], was reduced in *Insl5*−/− mice after a 12 h fast. We observed that glucose production from pyruvate was delayed and slightly reduced in *Insl5*−/− mice compared with WT mice (Figure 3F); this alteration in pyruvate tolerance suggests that hepatic glucose production may be compromised in *Insl5*−/− mice.

We next investigated whether *Insl5* deficiency modulated the protein levels of G6Pase and PEPCK, key enzymes in the gluconeogenic process, in the liver of mice fasted for 6 or 12 h. Although no changes were observed after a 6 h fast (data not shown), G6Pase protein levels and activity were significantly reduced in *Insl5*−/− mice compared with WT counterparts after a 12 h fast (Figure 3G,H). PEPCK levels were not altered by *Insl5* deficiency (Figure 3I). We did not observe any changes in gene expression of *G6pase* and *Pepck* in the kidney, another organ that is able to perform gluconeogenesis [29], [30], between *Insl5*−/− and WT mice (Supplementary Figure 3A,B).

Glucose is stored as glycogen in the liver, which is rapidly converted back to glucose in the absence of food. Thus, gluconeogenesis and glycogenolysis are essential for maintaining blood glucose at homeostatic levels [31]. We therefore investigated whether *Insl5* deficiency affected glycogen levels by analyzing liver tissue from fasted *Insl5*−/− and WT mice. After a 6 h fast, glycogen levels were significantly lower in *Insl5*−/− mice compared with WT mice, whereas both genotypes had exhausted their glycogen stores after a 12 h fast (Figure 3J). Glycogen levels were similar in *ad libitum* fed and re-fed *Insl5*−/− and WT mice (Supplementary Figure 3C).

These data suggest that *Insl5*−/− mice are compromised in their ability to perform hepatic glucose production and possess an altered glycogen metabolism.

## 4 Discussion

Here we showed that GF mice and antibiotic-treated CONV-R mice have elevated colonic expression of *Insl5* whereas expression of *Insl5* was higher in the brain of CONV-R versus GF mice. In agreement with our hypothesis that the elevated *Insl5* expression in colon of GF mice is due to reduced energy availability, we showed that *Insl5* expression is suppressed by colonization or by a high-fat diet in GF mice. Although both *Insl5* and *Gcg* are expressed in L-cells and are similarly regulated, we identified distinct functions for INSL5 and GLP-1. However, both colonic expressed peptides may confer adaptive responses to energy deficiency, and we propose that INSL5 acts as a sensor of energy and modulator of homeostatic glucose production under conditions of calorie restriction. Thus, we hypothesize that colonic L-cells respond to energy deficiency by: (1) increasing GLP-1 to reduce small intestinal transit [21] and thus increase nutrient absorption; and (2) simultaneously secreting INSL5 to increase hepatic glucose production.

Colonocytes utilize the bacterially produced butyrate as their primary energy source, and it has previously been reported that the microbiota has a greater impact on energy homeostasis in the colon than in other tissues [20]. Here we showed that increases in energy availability, induced by either the microbiota or dietary changes that circumvented the requirement of microbial fermentation for energy generation, suppressed colonic *Insl5* expression, similar to our previous observation showing energy-related regulation of *Gcg* expression [21]. Our results are consistent with an earlier study showing that plasma levels of INSL5 are high in the fasting state and reduced by feeding in mice [15] and suggest that INSL5 may act as a low-energy sensor. We attempted to measure circulating INSL5 in serum from GF and CONV-R mice using ELISA, but the signals were nonspecific; this issue has previously been reported [15].

To identify a potential role of INSL5 under conditions of energy restriction, we investigated the phenotype of *Insl5*-deficient mice. We showed that gut transit was not affected by *Insl5* deficiency. In addition, we did not observe any effect of *Insl5* deficiency on food intake after 12 h fast, in contrast to an earlier study by Grosse et al. reporting that INSL5 is an orexigenic hormone [15]. However, Grosse et al. tested the effect of injecting a bolus dose of INSL5 into mice [15], and it is likely that an acute increase in the levels of INSL5 will not have the same effect as the chronic *Insl5* deficiency in the mice used in our study. The lack of change in feeding behavior in *Insl5*−/− mice could be due to redundant signaling systems and potentially adaptive responses. Another possibility is that changes in feeding behavior may not be observed when an appetite hormone is removed but only when its levels are increased, as has been previously reported for GLP-1 [32].

In agreement with a report by Burnicka-Turek et al. showing that *Insl5*−/− mice at 3 and 9 months of age have impaired glucose homeostasis [13], we observed that *Insl5*−/− mice had impaired tolerance to glucose administered intraperitoneally. However, we did not observe any difference in oral glucose tolerance between *Insl5*−/− and WT mice. The different responses to the two glucose tolerance tests may be explained by the fact that oral but not intraperitoneal glucose administration activates the parasympathetic gut–brain axis, which is associated with increased glycogen storage and prevents glycogenolysis [33], [34], [35], [36], [37], [38], [39], [40]. Glycogen levels were similar in fed *Insl5*−/− and WT mice but were decreased to a greater extent in *Insl5*−/− mice compared with WT mice after a 6 h fast, in support of increased glycogen degradation in *Insl5*−/− mice, which may contribute to the increased glycemia following intraperitoneal glucose administration. Impairment in intraperitoneal glucose tolerance while exhibiting normal oral glucose tolerance was recently demonstrated in pancreas-specific GLP-1 receptor-deficient mice [41], further emphasizing that the body modulates glucose differently depending on route of delivery.

It is not clear why the fasting glucose levels in *Insl5*−/− mice were higher than in WT mice before the intraperitoneal glucose tolerance test but not before the oral glucose tolerance test or the pyruvate tolerance test. However, the intraperitoneal glucose tolerance test was performed one week after the oral glucose tolerance test in a subset of the same mice. Thus, the intraperitoneal glucose tolerance test was the second procedure in these mice whereas the other tests were not preceded by an earlier procedure. A potential explanation therefore could be that *Insl5*−/− mice are more sensitive to stress hormones (catecholamines), which activate hepatic glucose production [42], [43], and thus respond differently to stress when anticipating a second procedure.

In contrast to the impaired intraperitoneal glucose tolerance test in *Insl5*−/− mice, we observed improved insulin tolerance in these mice. Since the half-life of insulin is only about 10 min, most of the insulin is cleared after 60 min in an insulin tolerance test. Consequently, the blood glucose levels at early time points after an insulin injection indicate the insulin sensitivity of the mouse whereas the glycemic difference observed at later time points generally reflects the counter-regulatory response (i.e., release of hormones such as glucagon and catecholamines) [44], [45]. Our data showed that glucose levels in the WT mice were not reduced any further after the 30 min time point but were still decreasing at this time point in the *Insl5*−/− mice and the lowest level was observed at 60 min (Figure 3E). However, after the 60 min time point, glucose levels in the *Insl5*−/− mice increased at a faster rate. These data could potentially indicate a delayed or impaired onset of counter-regulatory responses in the *Insl5*−/− mice.

We hypothesized that the altered counter-regulatory responses in *Insl5*-deficient mice may result in reduced hepatic glucose production under conditions of low blood glucose or after an overnight fast. We found that *Insl5*−/− mice displayed a reduction in hepatic glucose production following a pyruvate tolerance test together with corresponding reductions in liver G6Pase protein levels and enzyme activity after a 12 h fast, which may suggest reduced gluconeogenesis. However, the difference in the pyruvate tolerance test between WT and *Insl5*−/− mice was small and is not likely to fully explain the larger difference observed in the insulin tolerance test between the two genotypes. Because we also observed reduced glycogen levels in *Insl5*−/− versus WT mice after a 6 h fast, we thus speculate that reduced availability of glycogen, and thus reduced glycogenolysis, together with reduced gluconeogenesis could potentially explain the delayed ability to counterbalance the reduction in blood glucose during an insulin tolerance test in *Insl5*−/− mice.

The INSL5 receptor Rxfp4 has been detected in a number of tissues including the liver and pancreas [7] and in myenteric neurons [15], and thus INSL5 may regulate hepatic gluconeogenesis and/or glycogenolysis directly or indirectly by modulation of glucagon or catecholamine levels or by signaling through neurons. Taken together, tissue specific knock-outs of *Insl5* may provide insights into whether the contrasting effects in glucose and insulin tolerance results from opposing effects of gut and brain produced INSL5. Interestingly, *Insl5* regulation by the microbiota is opposite in brain and gut.

In summary, we have shown that colonic expression of *Insl5* is regulated by the microbiota and energy availability. Our findings suggest that INSL5 is a new hormone that promotes hepatic glucose production, although its effect is mild. We propose that INSL5 may act as a sensor of energy and modulator of homeostatic glucose production under conditions of energy deprivation.

## Appendix A Supplementary data

Figure S1 **INSL5 is expressed in L-cells. (A)** INSL5 (green) co-localizes with GLP-1 (red) immunoreactive colonic cells, and appears yellow in the merged panel in cells from C57Bl/6 WT but not *Insl5*−/− mice. **(B)** INSL5 (green) co-localizes with PYY (red) immunoreactive colonic cells, and appears yellow in the merged panel in cells from C57Bl/6 WT but not *Insl5*−/− mice. (20× magnification, scale bar is 200 μm).

Figure S2 **Characterization of the C57Bl/6 *Insl5*−/− mouse. (A)** Body weight of C57Bl/6 WT and *Insl5*−/− mice from weaning until 30 weeks of age (*n* = 18–20). **(B)** Total body fat (*n* = 7–9), **(C)** epididymal fat pad weight (*n* = 7–9), **(D)** food intake (for 1 h after a 12 h fast) (*n* = 21) and **(E)** gut transit (*n* = 9–12) in C57Bl/6 WT and *Insl5*−/− mice. **(F)** Intraperitoneal glucose tolerance test (IPGTT), data are normalized to baseline levels of glucose. Data are mean ± SEM. *p < 0.05, **p < 0.01, ***p < 0.001.

Figure S3 **Gluconeogenic gene expression is not altered in kidney**. **(A)***G6pase* and **(B)***Pepck* expression in kidney of C57Bl/6 WT and *Insl5-/-* mice (*n* = 8−10). **(C)** Glycogen levels in liver of *ad lib* fed (fed) and re-fed C57Bl/6 WT and *Insl5-/-* mice (*n* = 5−6). Data are mean ± SEM.
