# Supplementary material for: Insulin-like peptide 5 is a microbially regulated peptide that promotes hepatic glucose production
Source: Mol Metab. 2016 Jan 25;5(4):263–70. doi: 10.1016/j.molmet.2016.01.007 (PMC4811983; doi:10.1016/j.molmet.2016.01.007)
Supplement: Supplementary file 1 [file mmc1.docx]

**Supplementary Table 1**

**qRT-PCR Primers DNA sequence 5’-3’**

L32 forward CCTCTGGTGAAGCCCAAGATC

L32 reverse TCTGGGTTTCCGCCAGTTT

Insl5 forward TGTGAAGCTCTGTGGCCTGG

Insl5 reverse GGTGCCTGTGGATCTCGAAC

Gcg forward AGGGACCTTTACCAGTGATGT

Gcg reverse AATGGCGACTTCTTCTGGGAA

Pyy forward ACGGTCGCAATGCTGCTAAT

Pyy reverse GACATCTCTTTTTCCATACCGCT
