# Supplementary figures and images for: Insulin-like peptide 5 is a microbially regulated peptide that promotes hepatic glucose production
Source: Mol Metab. 2016 Jan 25;5(4):263–70. doi: 10.1016/j.molmet.2016.01.007 (PMC4811983; doi:10.1016/j.molmet.2016.01.007)

# Supplementary Figure 1

A

WT

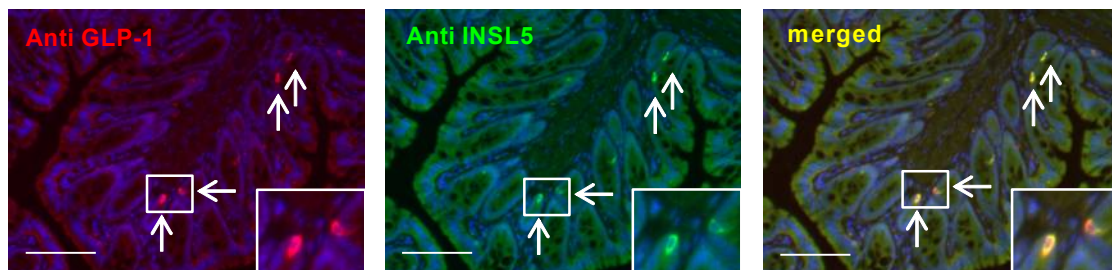

*Ins15<sup>-/-</sup>*

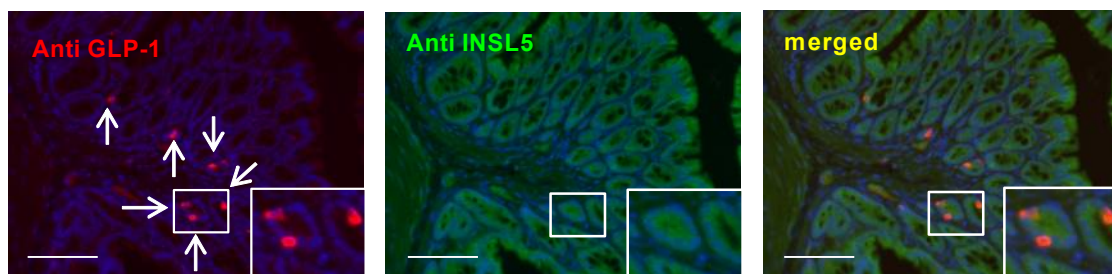

B

WT

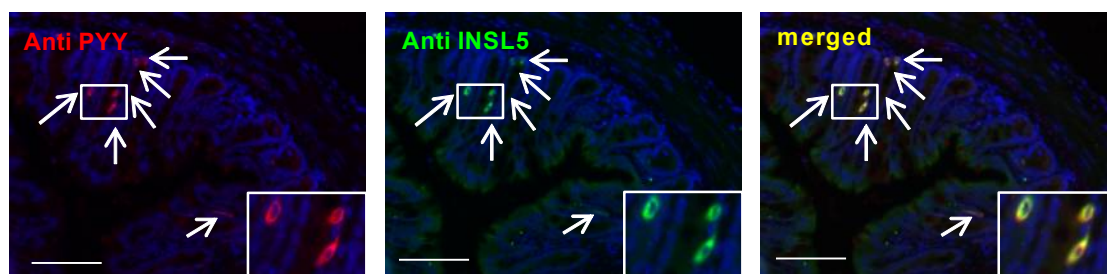

*Ins15<sup>-/-</sup>*

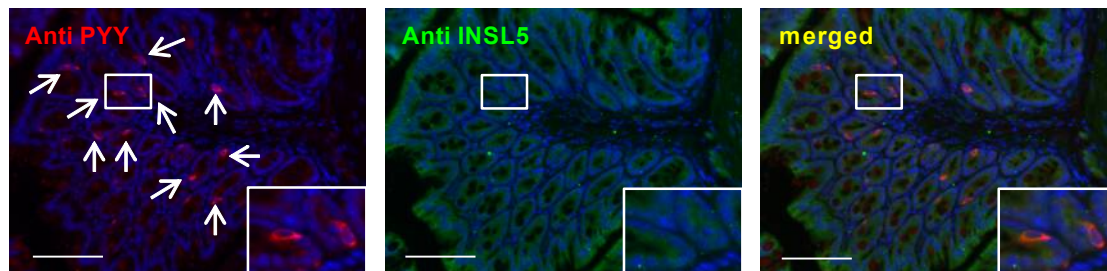

Supplement: Figure S1 — INSL5 is expressed in L-cells. (A) INSL5 (green) co-localizes with GLP-1 (red) immunoreactive colonic cells, and appears yellow in the merged panel in cells from C57Bl/6 WT but not Insl5−/− mice. (B) INSL5 (green) co-localizes with PYY (red) immunoreactive colonic cells, and appears yellow in the merged panel in cells from C57Bl/6 WT but not Insl5−/− mice. (20× magnification, scale bar is 200 μm). [file mmc2.pdf]

# Supplementary Figure 2

**A**

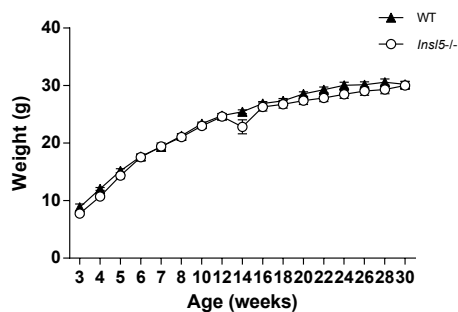

**B**

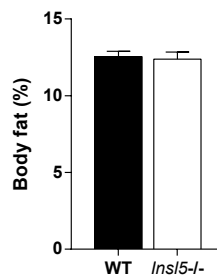

**C**

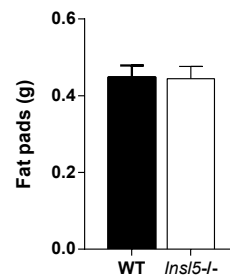

**D**

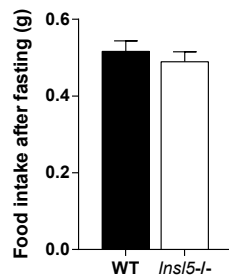

**E**

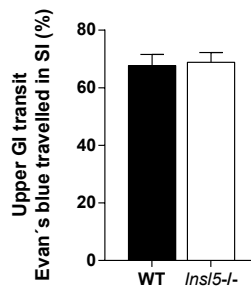

**F**

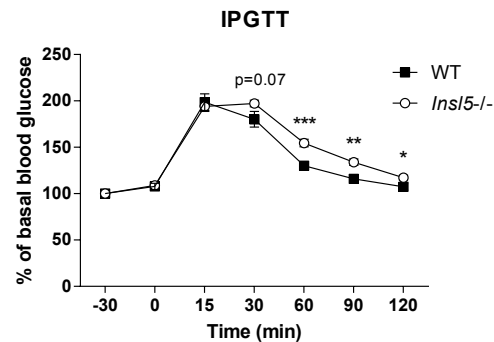

Supplement: Figure S2 — Characterization of the C57Bl/6 Insl5−/− mouse. (A) Body weight of C57Bl/6 WT and Insl5−/− mice from weaning until 30 weeks of age (n = 18–20). (B) Total body fat (n = 7–9), (C) epididymal fat pad weight (n = 7–9), (D) food intake (for 1 h after a 12 h fast) (n = 21) and (E) gut transit (n = 9–12) in C57Bl/6 WT and Insl5−/− mice. (F) Intraperitoneal glucose tolerance test (IPGTT), data are normalized to baseline levels of glucose. Data are mean ± SEM. *p < 0.05, **p < 0.01, ***p < 0.001. [file mmc3.pdf]

# Supplementary Figure 3

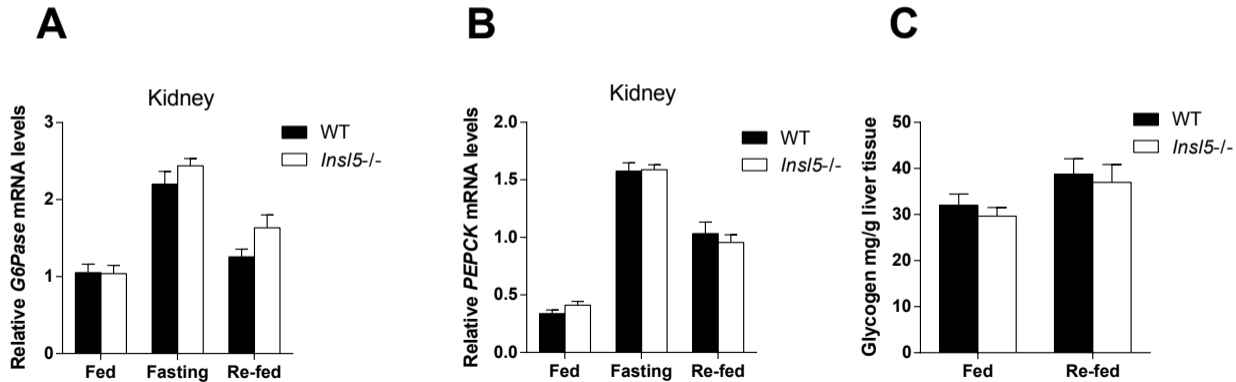

Supplement: Figure S3 — Gluconeogenic gene expression is not altered in kidney. (A)G6pase and (B)Pepck expression in kidney of C57Bl/6 WT and Insl5-/- mice (n = 8−10). (C) Glycogen levels in liver of ad lib fed (fed) and re-fed C57Bl/6 WT and Insl5-/- mice (n = 5−6). Data are mean ± SEM. [file mmc4.pdf]
